# Luxation traumatique négligée de la hanche traitée par arthroplastie totale de la hanche: à propos de 2 cas

**DOI:** 10.11604/pamj.2015.20.313.4325

**Published:** 2015-03-31

**Authors:** Bensaad Soufiane, Hammou Naserddine, Mechchat Atif, El Ibrahimi Abdelhamim, Shimi Mohemmed, Elmrini Abdelmjide

**Affiliations:** 1 Service de Chirurgie Ostéoarticulaire B4 CHU, Hassan II, Fez, Maroc

**Keywords:** Luxation négligée, nécrose, coxarthrose, prothèse totale de la hanche, Neglected dislocation, necrosis, coxarthrosis wiki, Total hip replacement

## Abstract

Les luxations négligées de la hanche sont des lésions exceptionnelles. Elle concerne généralement des traumatismes graves, où les lésions ostéo-articulaires ne sont pas minutieusement recherchées, ou relayer au second plan. L’évolution inéluctable se fait vers la nécrose de la tête fémorale et la coxarthrose. L'arthroplastie permet de raccourcir cette évolution et de réinsérer rapidement ces patients.

## Introduction

Les luxations négligées de la hanche sont des lésions des traumatismes graves où les lésions ostéo-articulairesne ne sont pas minutieusement recherchées. L’évolution est marquée par la nécrose de la tête fémorale et la coxarthrose. L'arthroplastie permet permet de ponter cette évolution. A travers deux observations nous décrivons les différents aspects cliniques, et radiologiques, pronostiques et thérapeutiques de cette pathologie.

## Patient et observation

### Observation numéro 1

**Figure 1:**
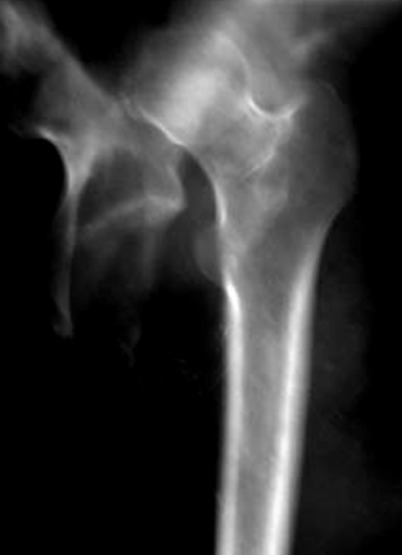
Luxation de la tête fémorale haute avec densification de la tête

**Figure 2:**
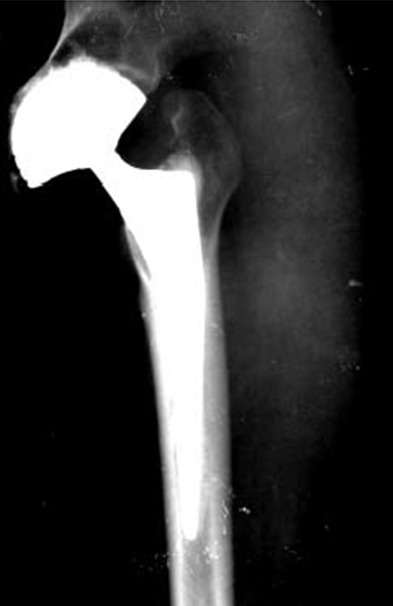
PTH double mobilité

A.R, patiente âgée de 38 ans, victime d'une chute de d'un lieu élevée, ayant occasionné un traumatisme de la hanche droite, associé à un traumatisme crânien grave responsable d'un coma. Le traumatisme de la hanche est resté négligée. Elle avait consulté deux années après pour raccourcissement du membre inferieur droit, douleur de la hanche avec un périmètre de marche de 5 min, avec une grosse boiterie. L'examen révélait une inégalité de longueur de 5 cm et une raideur de la hanche. La mobilisation était douloureuse. Le moyen fessier était à 2, le Trendelenburg était positif. La radiographie standard montrait une luxation haute non appuyée de la tête fémorale, qui était manifestement nécrosée (Figure 1). La patiente a été opéré par voie d'abord postéro-externe de Moore, la réduction avait nécessité une grande libération du moyen fessier, pour éviter la trochanterotomie et la ténotomie des adducteurs. Le cotyle était comblé de fibrose. La mise en place d'une prothèse cimentée à double mobilité était décidée pour éviter l'instabilité post-opératoire relative à la rétraction musculaire. Les suites postopératoires étaient simples, le contrôle postopératoire était bon (Figure 2). Actuellement les deux membres sont isolongs, la marche est stable, sans aide externe, indolore et la mobilité quasi normale. Le score de Haris est de 96, après un recule de 18 mois.

### Observation numéro 2

**Figure 3:**
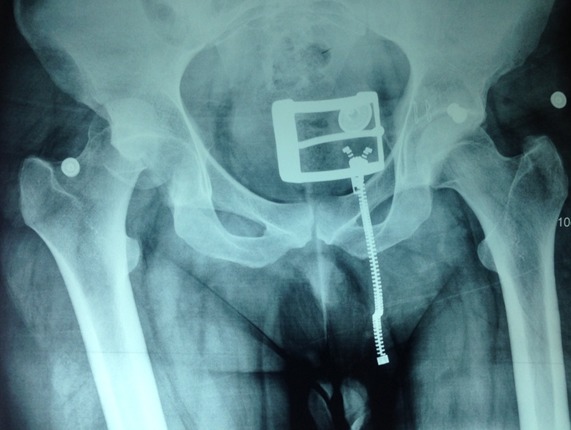
Luxation de haute de la tête fémorale

**Figure 4:**
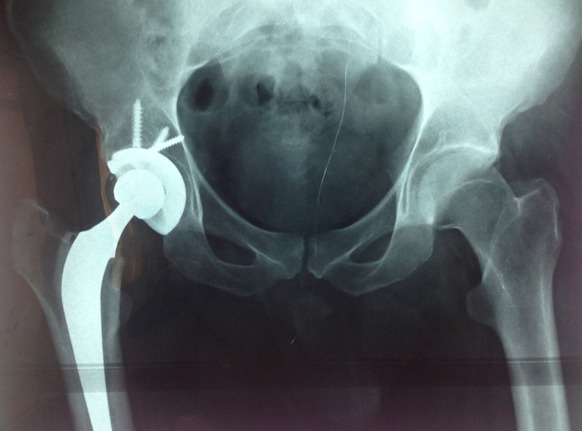
PTH hybride (tige cimentée, cotyle vissée à hydroxi appatite)

E.A, patient de 28 ans, victime d'un accident de la voie publique, occasionnant chez lui un traumatisme de la hanche droite associé à un traumatisme crânien grave obligeant le patient à séjourner en réanimation. Le traumatisme de la hanche est resté négligée. Quarante cinq jours après le traumatisme et devant la persistance d'une impotence fonctionnelle totale du membre inférieur droit le patient a consulté. L'examen clinique a objectivé un raccourcissement du membre inférieur droit de 3 cm, une raideur de la hanche droite et une mobilité douloureuse de cette hanche. La radiographie standard montrait une luxation haute non appuyée de la tête fémorale associée à une fracture de la paroi postérieure du cotyle (Figure 3). Le patient a été opéré par voie d'abord postéro-externe de Moore, avec mise en place d'une prothèse totale de la hanche hybride avec tige fémorale cimentée et cotyle à hydroxi apatite vissée. Les suites postopératoires étaient simples, le contrôle postopératoire était bon (Figure 4). Les deux membres sont isolongs, la marche est stable, sans aide externe, indolore et la mobilité quasi normale. Le score de Haris est de 95, après un recule de 22 mois.

## Discussion

Les luxations postérieures négligées de la hanche sont l'apanage de polytraumatisé où les lésions de l'appareil locomoteur passent inaperçues ou sont relayées au second plan [1]. Le risque de nécrose de la tête fémorale et d'arthrose est majeur [2–4], d'autant que la lésion est ancienne [5, 6]. Le pronostic est meilleur chez l'enfant [7]. L'association à une fracture de la tête fémorale amplifie le risque de nécrose avasculaire. Dans plusieurs séries de luxations postérieures négligées de la hanche, toute les fractures luxation de types 5 d'Hepstein réduites entre 3ème jour et 3ème mois, avaient un mauvais pronostic et une arthroplastie de 1ère intention est recommandée [8]. Nous avons adopté cette attitude d'autant sue la lésion était ancienne de 45 jours. D'autres rapportent de bons résultats, par traction continue, après 6mois à 3 ans de recul [9]. Dans une étude de réduction avec ostéosynthèse des fractures luxations, Letournel avait constaté la nécrose dans 2 cas sur 3 [10]. Le pronostic est d'autant plus réservé qu'il s'agit d'un polytraumatisme, l’état de choc altère d'avantage la perfusion de la tête fémorale [11]. La mise en place d'une arthroplastie nous parait plus réaliste en vue de toutes ces données, pour éviter cette évolution grevée d'un haut risque de nécrose et d'arthrose. La lésion du nerf sciatique, est un risque décrit dans 0 à 20% des cas. Mais le risque de lésion iatrogénique, existe dans la chirurgie prothétique en raison des modifications de repères anatomiques et de la fibrose prés du cotyle, parfois engainant le nerf. Son repérage est d'une importance capitale pour éviter de le léser en per-opératoire.

## Conclusion

La luxation traumatique de la hanche doit être réduite en urgence. Chaque heure qui s’écoule, aggrave le pronostic de la hanche. Les luxations invétérées doivent être traitées d'emblée par arthroplastie, pour réinsérer sans délai ces patients longtemps exclus de la vie active.
